# Necrology

**Published:** 1844-06

**Authors:** 


### NECROLOGY.

Dr. Francis W. Campbell, of Spring Hill, Arkansas, died at Jackson, Tennessee, on the 5th of April last, of abscess of the liver, resulting from repeated attacks of bilious fever. The abscess discharged its contents into the cavity of the chest—whether into the pleura or the lungs, is not stated. Dr. C. was 35 years of age, a skilful physician, a gentleman and a christian, whose loss will be keenly felt by a large circle by whom he was highly and justly esteemed. Y.

